# Health-related quality of life and influencing factors among rural left-behind wives in Liuyang, China

**DOI:** 10.1186/1472-6874-14-67

**Published:** 2014-05-14

**Authors:** Jinyao Yi, Bin Zhong, Shuqiao Yao

**Affiliations:** 1 Medical Psychological Institute, Second Xiangya Hospital, Central South University, NO139 Renmin Road, Changsha 410011, China; 2 Key Laboratory of Psychiatry and Mental Health of Hunan Province, Central South University, NO 139 Renmin Road, Changsha 410011, P R China; 3 Hunan Province Technology Institute of Psychiatry, Central South University, NO 139 Renmin Road, Changsha 410011, P R China

**Keywords:** Left-behind wives, Rural area, Health-related quality of life, Cross-sectional study

## Abstract

**Background:**

In China, the number of left-behind wives in rural areas has reached 47 million. Left-behind wives might have more psychological stress and lower life quality. This study was to examine the health-related quality of life and influencing social and cognitive factors in a sample of left-behind wives in rural areas of China.

**Methods:**

The demographic data questionnaire, the Short Form 36 Health Survey Scale, Center for Epidemiological Studies Depression Scale, Perceived Social Support Scale, Simplified Coping Style Questionnaire, and Perceived Stress Scale were completed by a sample of 1,893 left-behind wives and 969 non-left-behind wives.

**Results:**

Left-behind wives had lower scores on physical component summary (PCS), mental component summary (MCS) , and all the eight subscales of the SF-36 than non-left-behind wives (*P* < 0.05), especially on the role limitations due to emotional problems (RE) and on MCS. Compared to non-left-behind wives, left-behind wives scored higher in depression, stress, and passive coping styles, and scored significantly lower in social support and active coping styles. Logistic regression analysis showed that the status of being left behind, age, education years, monthly income, employment, physical health status, active coping styles, and depression were influencing factors on the PCS of rural women, whereas the status of being left behind, monthly income, physical health status, sense of marriage security, stress, social support, passive coping styles, and depression were influencing factors on the MCS of rural women.

**Conclusions:**

Left-behind wives scored lower on health-related quality of life than non-left-behind wives. Low health-related quality of life was associated with left behind status, older age, less education, low monthly income, unemployment, bad physical health status, passive coping styles, low social support, high level of stress, and high depression.

## Background

Women whose husbands have migrated in search of work are referred to as left-behind wives, wives left behind, or left-behind women [1]. In China, the title of "left-behind wives" refers to rural female residents who are left behind by their migrant husbands for the sake of business or other employment opportunities in places outside of their registered households for over 6 months at a time or more than 6 months over a year [2]. A study on left-behind wives in rural areas conducted in 2008 found that the number of left-behind wives in rural areas has reached 47 million [3].

The quality of a woman’s life is significantly affected by the environment surrounding her permanent residence [4]. A previous study showed that the poorest quality of life was found in permanent country dwellers, and that permanent residence in the country was an independent predictor of a poorer quality of life [4]. Shin and colleagues found that depression, health perception, social support, stress, and economic level were related to the quality of life of middle-aged women in rural Korea [5].

In China, as husbands have migrated away from home in search of job opportunities, the left-behind wives must take on the responsibilities of intensive labor, such as growing farm crops, running family chores, and taking care of seniors at home. These responsibilities, along with raising children and a sparse sex life, are causing left-behind rural wives more psychological stress and affecting their mental health [6]. However, left-behind wives have not yet been compared to their non-left-behind counterparts in health-related quality of life. In addition, little is known about the psychological and social factors that may impact a left-behind wife’s quality of life.

We sampled a group of rural left-behind wives living in Liuyang of China, to learn more about their quality of life and relevant psychological and social influencing factors. We made the following hypotheses:

First, compared with non-left-behind wives, left-behind wives would have worse health-related quality of life, less social support, and less active coping styles. Additionally, we hypothesized that they may be more depressed, more stressed, and use more passive coping styles.

Second, we hypothesized that compared to non-left-behind wives, left-behind wives would have worse physical health-related quality of life, which may be related to education, number of children, monthly income, sense of security of marriage, employment, physical health status, depression, stress, social support, and coping styles.

Our third hypothesis was that compared to non-left-behind wives, left-behind wives would have worse mental health-related quality of life, which may be related to education, number of children, monthly income, sense of marriage security, employment, physical health status, depression, stress, social support, and coping styles.

## Methods

### Participants

We chose to study residents of Liuyang of Hunan Province because it is within a medium level of economic development in China. More than 90% of its residents are living in rural areas; therefore, it is a representative rural area in China. We used multistage random sampling by first classifying all counties into high, medium, and low economic development, and then randomly selecting a county from each economic development level. In this way, the following three counties were selected: Yong’an, Sankou, and Shegang. Finally, six villages were selected at random (two from each of the three counties).

Of the 2,000 left-behind wives asked to participate, 1,893 (94.7%) volunteered. Women who declined to enroll did not differ significantly in age, education, or economic condition from those who volunteered. Additionally, 1,000 non-left-behind wives in the same villages were also asked to participate. Of these, 969 (96.9%) agreed to enroll. No significant differences were found in age, length of education, number of children, monthly income, sense of security of marriage, employment, or physical health status between the left-behind and non-left-behind groups (Table 1).

**Table 1:** Demographic data for left-behind wives and non-left-behind wives

**Characteristics**	**Left-behind wives *(n =*** **1893)**	**Non-left-behind wives *(n =*** **969)**
**Age**	36.48 ± 7.64	36.00 ± 8.81
**The length of education**	8.57 ± 2.65	8.76 ± 2.67
**Number of children**	1.44 ± 0.64	1.47 ± 0.71
**Monthly income (%)**		
Low (<1500RMB)	17.0	15.0
Middle (1500 ~ 4000RMB)	64.2	63.4
High (>4000RMB)	18.8	21.6
**Sense of security of marriage (%)**		
Very secure	13.3	16.8
Secure	56.2	55.4
General	24.2	22.3
Unsecure	5.3	4.4
Very unsecure	1.0	1.1
**Employment (%)**		
Working	43.3	46.1
Not working	56.7	53.9
**Physical health status (%)**		
Disease-free	74.3	76.1
Minor disease	24.7	23.0
Major diseases	1.0	0.9

*Note:*$\bar{\chi }\pm \text{SD}$*was used for continuous variables.*

This study was approved by the ethics committee of Central South University, and all participants provided written informed consent at the time of enrollment.

### Measures

#### Demographic data

The following demographic variables were assessed in a self-report questionnaire: age, years of education, number of children, monthly income, sense of security of marriage, employment, and physical health status.

#### Short form 36 health survey scale (SF-36)

The SF-36 survey is a self-administered, 36-item questionnaire designed to estimate health-related functions in eight distinct (subject) domains: physical functioning (PF); role limitations due to physical problems (RP); role limitations due to emotional problems (RE); vitality (VT); bodily pain (BP); social functioning (SF); mental health (MH); and general health perceptions (GH). After summing the items in the SF-36 survey, each scale is then standardized. The possible score in each subscale ranges from 0 to 100, with 0 indicating the lowest level of functioning and 100 indicating the highest. The eight subscales are hypothesized to form two distinct higher-order factors: physical and mental. The PF, RP, BP, and GH subscales are hypothesized to define the physical factor (PCS: physical component summary), while the MH, RE, SF, and VT scales are hypothesized to define the mental factor (MCS: mental component summary). The questionnaire was culturally adapted and translated into Chinese. It has been shown to possess high internal consistency and excellent reliability [7,8].

#### Perceived stress scale (PSS)

As a widely used global measurement of stress, the PSS consists of 10 items on a 5-point Likert scale [9]. Items measure how unpredictable, uncontrollable, and overloaded participants evaluate their lives during the last month. The questionnaire was culturally adapted and translated into Chinese. It has been reported to have high internal consistency and excellent reliability [10].

#### Perceived social support scale (PSSS)

The PSSS was used to evaluate the role of social support as an independent determinant of level of distress. The PSSS is composed of 12 items scored on a 7-point Likert scale with 1 = very strongly disagree and 7 = very strongly agree. Three subscale scores were calculated for Family, Friends, and Significant Other, along with a total score. Considering the goals of the current study, only the total was used. A low score in the PSSS suggests low social support [11]. The Cronbach’s α of the total scale is 0.88.

#### Simplified coping style questionnaire (SCSQ)

Coping style was assessed by the SCSQ. The SCSQ is a 20-item scale with scores ranging from 0 to 3 on each item. The SCSQ measures two coping styles: active coping (AC) and passive coping (PC). Active coping is used to handle the problem arousing emotional distress, whereas passive coping is used to handle the distressing emotions caused by the problem [12]. Questions 1 to 12 measure active coping strategies, and questions 13 to 20 measure passive coping strategies. The scale has shown high internal consistency for both active coping styles (Cronbach’s α = 0.89) and passive coping styles (Cronbach’s α = 0.78) [13].

#### Center for Epidemiological Studies Depression Scale (CES-D)

The CES-D is a 20-item scale that estimates depressive symptoms in the general population [14]. Each item is targeted at one symptom. Participants grade the occurrence of each symptom during the last week on a scale of 0 (rarely) to 3 (most of the time). Total scores are summed and range from 0 to 60. Higher scores indicate a higher degree of depressive symptoms. The translated Chinese version of the CES-D has shown a high degree of reliability and validity [15].

#### Data analysis

The SPSS 17.0 was used for statistical analyses. Categorical variables were expressed as frequency and percent distribution, while continuous variables were presented as means ± standard deviation. Normality assumptions were satisfied for continuous variables in this study. Chi-squared or Independent *t*-tests were used to evaluate the differences in demographic makeup and the SF-36, PSS, PSSS, SCSQ, and CES-D scores between left-behind wives and the control group (non-left-behind wives), and the Cohen d effect sizes were calculated to indicate the standardised mean difference between the two groups. Statistical significance of all two-tailed tests was set at *p* < 0.05.

Logistic regression analyses were used to predict the factors that influence the quality of life of rural left-behind wives. Based on the test score distribution report, physical and mental health composite scores of the SF-36 questionnaire were converted into dichotomous variables (scores ≥ 25% of the total were designated as 1; else, 0) [16], which served as dependent variable, respectively. Demographic parameters were given the following scores: whether rural wives are left-behind (left-behind = 1; non-left-behind = 0), age, years of education, household monthly income (low = 1; middle = 2; high = 3), marriage security (very secure = 1; secure = 2; moderately secure = 3; insecure = 4; very insecure = 5), employment (employed = 1; unemployment = 0), and health condition (no disease = 1; average = 2; major disease = 3). In addition, the number of children and CES-D, SCSQ, PSSS, and PSS scores served as independent variables and were analyzed for each factor’s influence on the physical and mental health composite scores.

## Results

### Comparison of SF-36 scores between left-behind wives and non-left-behind wives

Left-behind wives scored lower on physical component summary (PCS), mental component summary (MCS) , and all the eight subscales of the SF-36 than non-left-behind wives (P < 0.005 for all items, Table 2). The Cohen’s d showed that the largest differences were on the RE subscale and on MCS (Table 2).

**Table 2:** **Comparison of SF-36 scores between left-behind wives and non-left-behind wives** ( $\bar{\chi }\pm \text{SD}$**)**

	**Left-behind wives *(n =*** **1893)**	**Non-left-behind wives *(n =*** **969)**	**|Cohen’s d|**
**PF**	86.35 ± 13.94	91.82 ± 11.70^**^	0.43
**RP**	59.57 ± 39.48	79.39 ± 35.39^**^	0.53
**BP**	70.32 ± 14.94	74.97 ± 15.51^**^	0.31
**GH**	59.15 ± 18.98	66.35 ± 18.11^**^	0.39
**VT**	63.79 ± 16.22	70.47 ± 15.45^**^	0.42
**SF**	76.87 ± 17.48	83.99 ± 15.66^**^	0.43
**RE**	55.54 ± 39.38	81.70 ± 30.23^**^	0.75
**MH**	62.74 ± 14.46	67.80 ± 14.53^**^	0.35
**PCS**	68.85 ± 16.60	78.13 ± 16.18^**^	0.57
**MCS**	64.73 ± 15.66	75.99 ± 13.04^**^	0.78

^**^Significant difference between left-behind wives and non-left-behind wives, P < 0.01.

PF: physical functioning; RP: role limitations caused by physical problems; BP: bodily pain; GH: general health perceptions; VT: vitality; SF: social functioning; RE: role limitations caused by emotional problems; MH: mental health; PCS: physical component summary; MCS: mental component summary.

|Cohen’s d|: absolute value of Cohen’s d.

### Comparison of depression, stress, social support, and coping style scores between left-behind wives and non-left-behind wives

Compared with non-left-behind wives, left-behind wives scored significantly higher in CES-D (depression), PSS (stress) and PC (passive coping), and scored lower in PSSS (social support) and AC (active coping) (*P* < 0.005 for all items, Table 3). However, the differences were small (all Cohen’s d < 0.50).

**Table 3:** **Comparison of psychological measures between left-behind wives and non-left-behind wives** ( $\bar{\chi }\pm \text{SD}$**)**

	**Left-behind wives *(n =*** **1893)**	**Non-left-behind wives *(n =*** **969)**	**|Cohen d|**
CES-D	10.90 ± 10.37	7.93 ± 10.15^**^	0.29
PSS	17.23 ± 4.22	14.89 ± 6.70^**^	0.42
PSSS	59.84 ± 11.92	62.78 ± 12.03^**^	0.25
AC	20.62 ± 6.70	23.22 ± 7.96^**^	0.35
PC	10.61 ± 5.84	9.53 ± 5.93^**^	0.18

^**^Significant difference between left-behind wives and non-left-behind wives, P < 0.01.

CES-D: center for epidemiological studies depression scale; PSS: perceived stress scale; PSSS: perceived social support scale; AC: active coping; PC: passive coping.

### Logistic regression of influential factors contributing to the quality of life

The logistic regression analyses showed that the status of being left behind was an independent predictor of low physical health. Older age, less education, low monthly income, unemployment, bad physical health status, high level of depression, and less active coping styles also predicted low physical health in rural women. The status of being left behind was an independent predictor of low mental health. Low monthly income, sense of insecure marriage, bad physical health status, high stress, less social support, more passive coping styles, and high depression also predicted low mental health in rural women (Table 4).

**Table 4:** Result of the logistic regression analyses on quality of life

	***βeta***	**S.E.**	**Wald**	***OR***	***OR *** **95.0% CI**		***P***
					**Lower**	**Upper**	
**Physical component summary**							
Left behind	-1.136	.115	97.716	.321	.256	.402	.000
Age	-.029	.006	20.482	.972	.960	.984	.000
Length of education	.042	.020	4.533	1.043	1.003	1.083	.033
Monthly income	.221	.080	7.605	1.247	1.066	1.459	.006
Employment	.235	.097	5.888	1.265	1.046	1.530	.015
Physical health status	-1.247	.097	164.644	.287	.237	.348	.000
Active coping styles	.018	.007	5.789	1.018	1.003	1.033	.016
Depression	-.035	.005	55.397	.966	.957	.975	.000
**Mental component summary**							
Left behind	-1.612	.126	163.960	.199	.156	.255	.000
Monthly income	.166	.081	4.225	1.181	1.008	1.384	.040
Marriage security	-.151	.062	6.001	.860	.762	.970	.014
Physical health status	-.709	.100	49.885	.492	.404	.599	.000
Stress	-.050	.011	19.346	.952	.931	.973	.000
Social support	.235	.098	5.707	1.264	1.043	1.533	.017
Passive coping styles	-.025	.008	9.042	.975	.959	.991	.003
Depression	-.048	.005	97.259	.953	.944	.962	.000

OR: odds ratios; CI: confidence intervals.

## Discussion

In the current study, the health-related quality of life and its influencing factors were investigated in1,893 left-behind and 969 non-left-behind wives in China. To our knowledge, this is the first study to investigate health-related quality of life in rural left-behind wives of China with a large sample size. Left-behind wives scored significantly lower on the eight subscales of SF-36, PCS, MCS, social support, and active coping styles and scored significantly higher on depression, stress, and passive coping styles compared to non-left-behind wives. With respect to physical health, the differences between left-behind wives and non-left-behind wives were larger on physical functioning (PF) and role limitations due to physical problems (RP) than on the other subscales. With respect to mental health, the differences between left-behind wives and non-left-behind wives were largest on the subscale of role limitations due to emotional problems (RE).

Research conducted by Ma et al. (2008) found that the quality of life for left-behind children is significantly lower than that of non-left-behind children [17]. Additionally, research conducted by Chen et al. [18], found that left-behind senior citizens from rural areas in Hunan, Hubei, Sichuan, and Guizhou provinces score low on quality of life [18]. The results of the above two studies, together with those of the current study, provide evidence that left-behind groups have a poorer quality of life compared to non-left-behind groups. As a greater number of rural labor forces are migrating away from their families, the left-behind groups must take on greater burdens. For example, left-behind wives must shoulder greater financial, societal, and family responsibilities and, thus, have a greater degree of stress and lower quality of life.

In the current study, age, education, monthly household income, employment, and physical status were influencing factors in the quality of life for rural women, regardless of left-behind status. Older left-behind rural women with lower education, unemployment, lower monthly household income, and worse physical status had lower quality of life. Prior studies have found that whether left behind or not [19], age [20], household financial condition [21], and physical status [22] are influencing factors on quality of life. The present results along with these prior studies should be taken into consideration when administrating to rural areas.

With respect to psychological and social factors, passive coping styles, social support, daily stress, and depression were influential factors to the quality of life for rural women. Programs aimed at increasing mental health awareness of rural women should be implemented. Mental health counseling and intervention systems should be improved to help rural women cope with stress effectively by adopting an active coping style and building a social support system, so as to improve their quality of life.

Two limitations of the current study should be noted. First, self-report measures were used to assess quality of life, stress, social support, coping styles, depressive, and anxiety symptoms, which were likely to be influenced by informant bias. More sophisticated methods of analysis, such as interviewing procedures that assess quality of life and stress level, along with depressive and anxiety symptoms may provide a more accurate assessment. Second, the sample used in the current study may not be generalizable to other populations. Future studies should examine similar hypotheses among rural left-behind wives in other Chinese provinces to determine if the current findings can be replicated.

## Conclusions

Left-behind wives had lower health-related quality of life than non-left-behind wives. Low health-related quality of life was associated with left behind status, older age, less education, low monthly income, unemployment, bad physical health status, passive coping styles, low social support, high level of stress, and high depression. Mental health counseling and intervention systems should be improved to help rural women cope with stress effectively by adopting an active coping style and building a social support system, so as to improve their quality of life.

## Abbreviations

SF-36: Short form 36 health survey scale; PSS: Perceived stress scale; PSSS: Perceived social support scale; SCSQ: Simplified coping style questionnaire; CES-D: Center for epidemiological studies depression scale; AC: Active coping; PC: Passive coping; PF: Physical functioning; RP: Role limitations caused by physical problems; BP: Bodily pain; GH: General health perceptions; VT: Vitality; SF: Social functioning; RE: Role limitations caused by emotional problems; MH: Mental health; PCS: Physical component summary; MCS: Mental component summary.

## Competing interests

The authors declare that they have no conflict of interests.

## Authors’ contributions

YS and YJ was involved in the design of the study. ZB was responsible for data collection. YS and ZB analyzed the data. YJ and ZB wrote the manuscript. All authors have read and approved the final version of the manuscript.

## Authors’ information

Jinyao Yi and Bin Zhong are co-first authors.

## Pre-publication history

The pre-publication history for this paper can be accessed here:

http://www.biomedcentral.com/1472-6874/14/67/prepub
